# The European Brain Council Value of Treatment studies in depression and autism

**DOI:** 10.1192/j.eurpsy.2023.2459

**Published:** 2023-12-05

**Authors:** Sophia Frangou

**Affiliations:** Icahn School of Medicine at Mount Sinai, New York, USA; Centre for Brain Health, University of British Columbia, Vancouver, Canada

European Psychiatry has partnered with the European Brain Council (EBC) to present a collection of studies that showcase the breadth of the work the Council supports. The EBC (https://www.braincouncil.eu/) is a network of scientific and professional societies, patient organizations, and commercial companies that support research aimed at improving the lives of Europeans suffering from brain disorders. In 2022, the EBC celebrated 20 years of service to the field, during which it has fostered discussions and collaborations to strengthen and harmonize European mental health research and care. This collection showcases the work undertaken as part of the EBC project on the Value of Treatment (VOT2), which was initiated in 2019. As clearly outlined by Simon et al. [1], the VOT2 project combines care pathways analysis and economic analysis to identify treatment gaps, evaluate potential outcomes and costs of optimized care, and offer policy recommendations in collaboration with the EBC’s scientific societies and patient organizations. The collection demonstrates the utility of the VOT2 research framework when applied to major depressive disorder (MDD) and autism spectrum disorders (ASD), two prevalent and disabling disorders.

Strawbridge et al. [2] highlight the findings of the EBC VOT2 study on adult MDD, which identified significant gaps in the care pathways for patients with MDD in Europe. A review of the relevant literature revealed that approximately 52% of MDD episodes go undiagnosed. Among those diagnosed, roughly 38% do not receive treatment, which increases to an average of 65% when considering samples inclusive of undiagnosed cases. The average delay in treatment is around 4 years (range: 1–8 years). Upon initiating treatment, about 66% of individuals receive follow-up, but often below the standards advocated by established guidelines. Only about 19% of those diagnosed with MDD access psychiatric care, and an even smaller percentage access specialized mood disorder care. The theme of gaps in care pathways was further pursued by Wong et al. [3] in the context of the COVID-19 pandemic. The authors found that information on the impact of the pandemic on treatment pathways for MDD was incomplete. Nevertheless, the available data indicated a pandemic-related decrease in treatment rates and access to care. Additionally, the clinical effectiveness of the digital interventions that were widely deployed has yet to be evaluated. McCrone et al. [4] provide an informed account of the cost-effectiveness of reducing treatment gaps in MDD. A decision-tree framework spanning 27 months was employed, tracing care pathways with different detection rates and treatment modalities for MDD. The corresponding costs and quality-adjusted life years (QALYs) were computed for Germany, Hungary, Italy, Portugal, Sweden, and the UK. Their model shows that reducing detection and treatment gaps to 50 and 25%, respectively, is associated with higher short-term costs but long-term cost-efficiency.

Mendez et al. [5] highlight the utility of the EBC VOT2 framework for studying care pathway gaps in children and adolescents with ASD living in Italy, Spain, and the UK. The authors used a combined approach involving a literature review and an online survey of 663 carers of youth with ASD (aged <18 years). Between a third to half of the respondents reported receiving no guidance or support after first expressing their concerns to a health professional and having to wait over a year for a specialist diagnostic appointment. Nearly half of the respondents reported long waits before being offered publicly funded treatment and a general lack of support for carers. In their second paper, Mendez et al. [6] extend the scope of inquiry to gaps in care pathways for youth (aged <18 years) with comorbid ASD and epilepsy living in Italy, Spain, and the UK. Epilepsy is present in approximately 7% of youth with autism and exacerbates the psychosocial disability of those youth who suffer from both disorders. However, based on the responses of the same group of carers as in the previous study, the authors found that only about a third of youth presenting with autism were screened for epilepsy. Moreover, the authors pointed out other gaps, including the lack of clear and harmonized guidelines regarding screening for epilepsy and the appropriate use of antiepileptic drugs. Tinelli et al. [7] examined the economic outcome 6 years after the completion of a particular type of early intervention assessed during the Preschool Autism Communication Trial (PACT). The intervention consisted of 12 sessions aimed at enhancing social communication in autistic children delivered over a period of 6 months. The PACT trial enrolled 74 children with ASD in the active arm and 69 children with ASD in the control arm which involved treatment as usual. The intervention improved the symptoms of ASD and family interactions, leading to cost-saving in terms of informal care.
